# The association between the Acute Physiology Score and sepsis in ICU non-surviving patients with inflammatory bowel disease: a retrospective cohort study from the eICU-CRD

**DOI:** 10.3389/fmed.2026.1761815

**Published:** 2026-06-03

**Authors:** Chenyang Li, Zhexuan Liu, Daniel D. Zhang, Fenghua Li, Dong Wang, Yuxin Luo, Yue Yao, Qian Liu, Xu Teng, Shuang Chen, Xiaolan Zhang

**Affiliations:** 1 Department of Gastroenterology, The Second Hospital of Hebei Medical University, Shijiazhuang, China; 2 Department of Physiology, Hebei Medical University, Shijiazhuang, China; 3 Department of Biology, University of California Riverside, Riverside, CA, United States; 4 Department of Pediatric Immunology and Infectious Disease Research Institute, Cedars Sinai Medical Center, Los Angeles, CA, United States

**Keywords:** Acute Physiology Score, inflammatory bowel disease, intensive care unit, risk factors, sepsis

## Abstract

**Background:**

Sepsis significantly contributes to ICU mortality in inflammatory bowel disease (IBD) patients. Early risk stratification remains challenging.

**Methods:**

This retrospective cohort study utilized the eICU-CRD database, analyzing 190 adult IBD patients who died during ICU admission. We employed weighted logistic regression, restricted cubic splines, and XGBoost to evaluate the association between Acute Physiology Score (APS) and sepsis.

**Results:**

Among 190 non-survivors (55 with sepsis), the sepsis group demonstrated significantly higher APS scores (49.71 ± 23.25 vs. 40.66 ± 18.78, *p* = 0.015). After full adjustment, APS remained independently associated with sepsis (OR = 1.027, 95% CI: 1.009–1.045, *p* = 0.004). Quartile analysis revealed a markedly elevated risk in the highest APS quartile (Q4 vs. Q1: OR = 4.856, 95% CI: 1.675–15.713, *p* = 0.005). XGBoost with SHAP analysis ranked APS as the second most important predictor. Subgroup analyses showed enhanced predictive performance in elderly (≥60 years) and male patients. APS positively correlated with anion gap (*p* = 0.044) and lactate (*p* = 0.018), and negatively with bicarbonate (*p* < 0.001).

**Conclusion:**

APS independently associated with sepsis in ICU non-surviving IBD patients. Given the small sample and study design, these findings are hypothesis-generating and restricted to this subgroup.

## 1 Introduction

IBD, comprising Crohn’s disease and ulcerative colitis, represents a significant global health burden with rising incidence worldwide (1, 2). Patients with IBD face substantially increased risks of critical illness, with sepsis emerging as a leading cause of intensive care unit (ICU) admission and mortality in this population (3, 4).

Sepsis is a life-threatening organ dysfunction caused by a dysregulated host response to infection (5), manifested as a systemic immune response that can progress to septic shock, multiple organ failure, and even death. Due to its severity and rapid progression, early detection and diagnosis of sepsis are crucial for timely intervention. Despite advances in organ support and a decreased incidence and related mortality (6), sepsis still results in approximately 11 million deaths globally each year (7), significantly impacting patient prognosis and healthcare services. Meanwhile, disease activity in IBD patients is significantly associated with infection risk, leading to a notably higher incidence of severe infections such as sepsis (8). Furthermore, prolonged use of steroids, immunosuppressants, biologics, or small molecules in IBD patients may exacerbate immunosuppression, thereby increasing the risk of opportunistic infections and sepsis (9, 10). Notably, patients with IBD have a 2–3 times higher risk of sepsis than the general population, and sepsis has become one of the leading causes of ICU admission and mortality in critically ill IBD patients (11, 12). Therefore, early risk stratification for sepsis in this high-risk population is particularly critical to improve clinical outcomes.

Upon ICU admission, the early prognosis of sepsis depends on the precise quantification of the patient’s physiological status, for which several scoring systems have been developed. Historically, the APACHE II score or Simplified Acute Physiology Score (SAPS II) (13) has been widely used, with the APACHE II score validated as an independent risk factor for predicting short-term mortality outcomes in adult sepsis patients admitted to the ICU. Notably, the APACHE II score has also been validated as independently relevant in studies predicting ICU mortality for IBD patients (4). The APS, based on objective and accurate physiological parameters, is relatively less influenced by immediate interventions and quantifies the severity of acute physiological disturbance by integrating 12 physiological variables (e.g., temperature, blood pressure, and arterial blood gases), and has been widely used to assess disease severity and prognosis in critically ill patients (14). Moreover, APS allows rapid and straightforward calculation of acute physiological instability. Given that the use of APS alone for assessing IBD patients has not been evaluated by any research, this study aims to investigate the association between APS and sepsis in non-surviving IBD patients admitted to the ICU, as a severity marker for the critically ill population.

## 2 Materials and methods

### 2.1 Data preprocessing

This study utilized a retrospective cohort design, drawing data from the eICU Collaborative Research Database (version 2.0). Notably, the eICU-CRD database is a large public database collaboratively created by Philips and the Massachusetts Institute of Technology (MIT) Laboratory for Computational Physiology (LCP), encompassing routine data from over 200,000 patients admitted to various ICUs in the United States in 2014 and 2015 (15). It collects a wealth of high-quality clinical data, including vital signs, nursing care plans, disease severity, diagnostic information, and treatment details. The eICU database used in this study is publicly available and can be accessed through registration (16).

### 2.2 Study population and inclusion/exclusion criteria

Data for this study were obtained from the eICU Collaborative Research Database (eICU-CRD, version 2.0), which contains inpatient data from multiple intensive care units (ICUs) across the United States during 2014–2015.

Inclusion criteria: (1) Diagnosis of inflammatory bowel disease (IBD) confirmed by ICD-9 codes: 555.9 (Crohn’s disease) or 556.9 (ulcerative colitis); (2) ICU length of stay <30 days and death occurring during the ICU admission.

Exclusion criteria: (1) Absence of IBD-related diagnostic data; (2) Missing data on ICU survival time or missing key physiological and laboratory parameters needed for APS calculation; (3) Missing baseline demographic or clinical data (e.g., age, sex, height, weight).

IBD: In this study, IBD was defined using ICD-9 codes recorded in the eICU-CRD database: 555.9 (Crohn’s disease) and 556.9 (ulcerative colitis). Due to the inherent structure of the public database, this study was limited to the above two IBD diagnostic codes, which were completely and uniformly recorded across multiple centers; more specific anatomical subtype codes (e.g., 555.0–555.8 and 556.0–556.8) could not be further incorporated. IBD is a group of chronic intestinal inflammatory disorders whose pathogenesis involves interactions between genetic and environmental factors, primarily affecting the digestive tract. Because this is a retrospective database analysis, no further clinical or endoscopic confirmation of specific IBD subtypes was performed (1). The study population comprised adult IBD patients identified by the aforementioned codes who died during their ICU admission, and the study conclusions are applicable exclusively to this specific population.

Sepsis: Sepsis was identified using ICD-9 codes recorded in the eICU-CRD database (2014–2015), which predates the 2016 Sepsis-3 consensus definition; therefore, sepsis was defined using administrative codes rather than the Sepsis-3 criteria. Sepsis cases were jointly identified through the following completely recorded ICD-9 codes available in the database: 038.9 (unspecified septicemia), 995.90 (systemic inflammatory response syndrome, unspecified), 995.91 (sepsis), 995.92 (severe sepsis), and 785.52 (septic shock). To minimize potential case under-ascertainment, patients with sepsis-related diagnoses recorded exclusively in comorbidity fields but without sepsis-related ICD codes in the primary admission diagnosis were additionally included. Due to limitations of the available data, this study could not distinguish whether sepsis was present at ICU admission or developed during the hospital stay. As with any case ascertainment based on ICD-9 codes, this approach has inherent limitations in sensitivity and specificity, which may introduce misclassification bias and limit the reproducibility of the findings.

### 2.3 Acute Physiology Score

APS is the physiological component of the APACHE II scoring system (13). It was calculated by summing the points assigned to the most abnormal values of 12 physiological variables within the first 24 h of ICU admission, as defined in the original APACHE II protocol (14). These variables include temperature, mean arterial pressure, heart rate, respiratory rate, oxygenation (PaO₂/FiO₂ ratio or A-aDO₂), arterial pH (or serum bicarbonate), serum sodium, serum potassium, serum creatinine (with adjustment for acute renal failure), hematocrit, white blood cell count, and the Glasgow Coma Scale (GCS) score. The APS quantifies the severity of acute physiological derangement, with higher scores indicating greater instability.

### 2.4 Covariates

Potential confounding factors were selected based on previous literature, including age (years), gender, height (cm), weight (kg), body mass index (BMI) (kg/m^2^), race, systolic blood pressure (mmHg), diastolic blood pressure (mmHg), heart rate (bpm), respiratory rate (bpm), peripheral oxygen saturation (%), hemoglobin (g/dL), platelet count (K/mcL), lymphocyte percentage (%), neutrophil percentage (%), red cell distribution width percentage (%), temperature (°C), anion gap (mmol/L), lactate (mmol/L), and bicarbonate (mmol/L). Age, gender, height, race, and weight were determined using the demographic variable file (DEMO) from the eICU-CRD survey, in which racial groups were categorized as African American, Asian, Caucasian, Hispanic, Native American, and others; gender was classified as male and female. Furthermore, patients were divided into an elderly group (aged ≥ 60 years) and a younger group (aged < 60 years). Laboratory data were determined based on the laboratory datasets from the eICU-CRD survey.

Body mass index (BMI) was calculated using height and weight recorded in the first 24 h after patient admission. BMI was subdivided into four groups according to World Health Organization (WHO) standard classifications: underweight (BMI < 18.5 kg/m^2^), normal weight (18.5 ≤ BMI<25 kg/m^2^), overweight (25 ≤ BMI<30 kg/m^2^), and obesity (BMI ≥ 30 kg/m^2^) (17).

### 2.5 Weighted logistic regression analysis

We performed weighted logistic regression to evaluate the association between the APS and the occurrence of sepsis among ICU non-survivors. APS was incorporated into the model both as a continuous variable and as a categorical variable to calculate odds ratios (ORs) with 95% confidence intervals (CIs). APS was divided into quartiles based on the interquartile range: first quartile (Q1), second quartile (Q2), third quartile (Q3), and fourth quartile (Q4), with the lowest quartile (Q1) serving as the reference.

Three statistical models were constructed. Model 1 was the unadjusted (crude) model. Model 2 was adjusted for weight and body mass index (BMI). Model 3 was further adjusted for diastolic blood pressure (DBP), systolic blood pressure (SBP), hemoglobin (HGB), lymphocyte percentage, anion gap, lactate, respiratory rate, and temperature based on Model 2. These covariates were selected based on clinical knowledge and prior critical care literature rather than by univariate statistical significance, in accordance with general principles that confounder selection should rely on substantive understanding of covariate-exposure and covariate-outcome relationships (18). Of note, temperature, respiratory rate, SBP, and DBP are integral components of the APS, while lactate, anion gap, and bicarbonate are downstream markers of the physiological derangement that the APS is designed to capture. Although variance inflation factor analysis indicated no critical statistical collinearity (all VIF < 10, Supplementary Table S4), the inclusion of these variables may conceptually constitute overadjustment and could attenuate the estimated association between APS and sepsis. Therefore, estimates from Model 3 should be interpreted with appropriate caution. To assess potential multicollinearity, we calculated the variance inflation factor (VIF) for all independent variables in Model 3; all VIF values were below 10, confirming the absence of substantial collinearity (Supplementary Table S4). Notably, temperature, respiratory rate, SBP, and DBP are integral components of the APS, while lactate, anion gap, and bicarbonate are downstream markers of the physiological derangement captured by the APS. Although the VIF analysis indicated no critical multicollinearity, the inclusion of these variables may theoretically introduce overadjustment, and this limitation is explicitly discussed in the Discussion section.

The weighting approach used in this study was not inverse probability of treatment weighting (IPTW), nor was any propensity score model constructed. The purpose of weighting was to enhance the stability and robustness of regression estimates given the small sample size, to balance baseline characteristic distributions between groups, and to account for the multicenter, stratified structure of the eICU-CRD database, thereby improving the representativeness of results across different ICUs. All non-normally distributed continuous covariates were transformed using weighted quartiles. Interaction analyses were performed to assess potential interaction effects between each subgroup and the APS. All statistical tests were two-sided, and *p*-values were adjusted using the false discovery rate (FDR). A *p*-value<0.05 was considered statistically significant.

### 2.6 Restricted cubic spline (RCS)

We used restricted cubic splines (RCS) with 4 knots (placed at the 5th, 35th, 65th, and 95th percentiles) to model the potential non-linear relationship between APS (as a continuous variable) and the log-odds of sepsis, adjusting for all covariates specified in Model 3.

### 2.7 eXtreme gradient boosting (XGBoost) model

In this study, the eXtreme Gradient Boosting (XGBoost) algorithm was employed for exploratory analysis of variable importance. The gradient boosting framework with decision tree integration was used to capture potentially complex nonlinear relationships between predictors and the outcome. A regularized objective function (incorporating L1/L2 penalty terms) was applied to enhance estimation stability and prevent overfitting.

Shapley Additive Explanations (SHAP) values were used as the core method to quantify the global importance of each variable and to visualize the directional impact of features on the risk of sepsis in patients with IBD, thereby improving the interpretability of the results. Variable importance was comprehensively evaluated based on feature contributions, with a primary focus on identifying key factors associated with sepsis and aiding in the interpretation of the mechanistic association between the APS and the outcome.

### 2.8 Clinical subgroup analysis

The association between APS and sepsis in non-survivors admitted to the hospital across different subgroups (age, gender, BMI) was evaluated through subgroup analyses. The subgroup and interaction effect analyses performed in this study evaluated whether the associations differed among various ages, genders, and BMI categories. Meanwhile, the visual comparison of effect sizes and confidence intervals (CIs) across multiple studies was performed using forest plots, which intuitively demonstrated the differences in odds ratios (ORs) and their 95% CIs between the two groups, facilitating a better understanding of the consistency and differences among various studies.

### 2.9 Sensitivity analysis

In this study, sensitivity analyses were performed to assess the robustness of the association. Specifically, we (1) recalculated the APS after excluding temperature and (2) recalculated the APS after excluding respiratory rate, then repeated the regression analyses to verify the stability of results.

### 2.10 Multiple imputation (MI)

To address missing data in baseline covariates, we performed multiple imputation using the MICE package in R, assuming a Missing at Random (MAR) mechanism. Five complete datasets were generated via chained equations, which incorporated all study variables and used appropriate imputation methods (predictive mean matching for continuous variables, logistic regression for binary variables). The resulting datasets were validated by comparing distributions before and after imputation. All subsequent analyses were performed on each imputed dataset, and the results were pooled using Rubin’s rules to derive final estimates. Normality of continuous variables was assessed using the Shapiro–Wilk test. Normally distributed variables were presented as mean ± standard deviation (SD), and non-normally distributed variables as median (interquartile range, IQR). Categorical variables were reported as counts and weighted percentages to account for the study weights inherent to the eICU-CRD database design.

### 2.11 Statistical methods

R software (version 4.4.3) was used for the processing and analysis of all data. This study was based on the eICU Collaborative Research Database (eICU-CRD), a multicenter critical care registry that includes sampling weights to enhance population representativeness. Therefore, categorical variables were described using counts and weighted percentages, with the weights directly adopted from the official analytical weights provided by the database, without additional calculation or adjustment.

Given the sample size limitations, this study employed a relatively simplified regression model for association analysis and did not introduce overly complex penalty terms or nonlinear models to avoid overfitting.

The Shapiro–Wilk test was used to assess the normality of all continuous variables. Normally distributed continuous variables were presented as mean ± standard deviation, while non-normally distributed continuous variables were presented as median (interquartile range, IQR). Categorical variables were presented as counts (percentages).

## 3 Result

### 3.1 Study subjects

**Figure 1:**
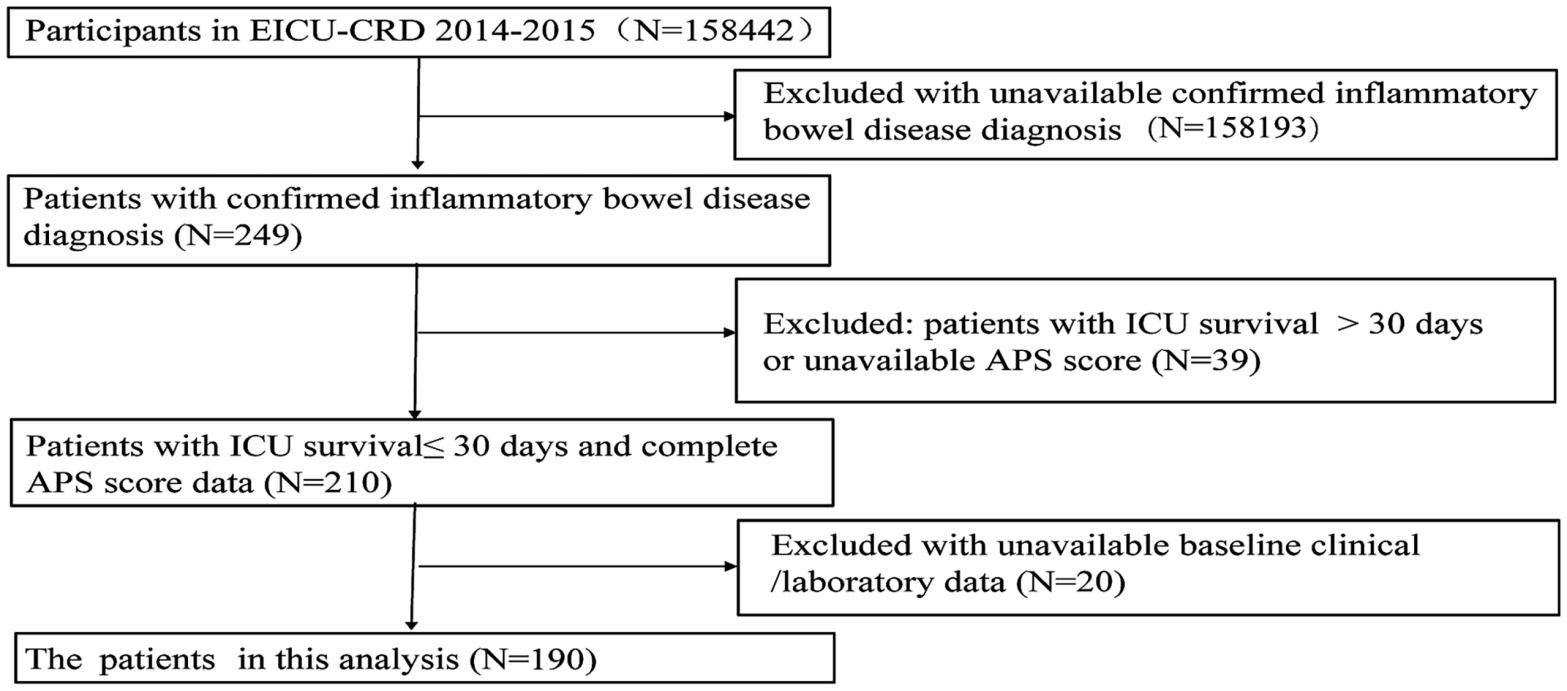
Study flow diagram of patient selection from the eICU-CRD. IBD was defined by ICD-9 codes 555.9 (Crohn’s disease) and 556.9 (ulcerative colitis). Baseline data included the 12 physiological parameters required for APS calculation plus height and weight for BMI.

Among the 190 IBD patients who died during ICU admission in this study, 55 had sepsis and 135 did not, as outlined in Figure 1. Of 158,442 patients, 158,193 were excluded due to the absence of a confirmed IBD diagnosis, leaving 249 patients with a confirmed IBD diagnosis. Among these, 39 patients were excluded because their ICU survival exceeded 30 days or their APS score was unavailable, leaving 210 patients. An additional 20 patients were excluded due to unavailable baseline clinical laboratory data. Ultimately, 190 patients were included in the analysis (55 with sepsis, 135 without sepsis), as outlined in Figure 1.

### 3.2 Baseline characteristics

Continuous variables were presented as mean ± standard deviation (for normally distributed data) or median (interquartile range, IQR) (for non-normally distributed data) based on their distribution types, and categorical variables were expressed as count (percentage). The Shapiro–Wilk test was used to assess the normality of continuous variables. A total of 190 ICU non-survivors were enrolled in this study, including 55 patients with sepsis and 135 patients without sepsis. Significant differences in baseline characteristics were observed between the two groups, as detailed below: (1) Weight: The weight of IBD patients with sepsis admitted to the ICU was significantly higher than that of the control group, with medians (IQR) of 77.80 (63.50, 97.24) kg and 72.20 (61.00, 86.80) kg, respectively (*p* = 0.014). (2) Diastolic Blood Pressure (DBP): The DBP of IBD patients with sepsis admitted to the ICU was significantly lower than that of the control group, with medians (IQR) of 55.00 (48.00, 69.00) mmHg and 66.00 (55.00, 76.00) mmHg, respectively (*p* = 0.002). (3) Systolic Blood Pressure (SBP): The SBP of IBD patients with sepsis admitted to the ICU was significantly lower than that of the control group, with medians (IQR) of 103.00 (88.00, 124.00) mmHg and 114.00 (101.00, 136.00) mmHg, respectively (*p* = 0.005). (4) Temperature: The temperature of non-survivors with sepsis admitted to the ICU was significantly higher than that of the control group, with medians (IQR) of 37.00 (36.60, 37.50) °C and 36.80 (36.40, 37.10) °C, respectively (*p* = 0.013). (5) Hemoglobin (HGB) Level: The HGB level of non-survivors with sepsis admitted to the ICU was significantly lower than that of the control group, with means ± standard deviations of 9.68 ± 2.15 g/dL and 10.42 ± 2.13 g/dL, respectively (*p* = 0.014). (6) Lymphocyte Percentage: The lymphocyte percentage of non-survivors with sepsis admitted to the ICU was significantly lower than that of the control group, with medians (IQR) of 8.00 (4.30, 13.60) % and 10.00 (6.00, 17.00) %, respectively (*p* = 0.031). (7) Anion Gap: The anion gap of non-survivors with sepsis admitted to the ICU was significantly lower than that of the control group, with medians (IQR) of 9.00 (7.00, 12.00) mmol/L and 10.00 (7.00, 14.00) mmol/L, respectively (*p* = 0.017). (8) Lactate: The lactate level of non-survivors with sepsis admitted to the ICU was significantly higher than that of the control group, with medians (IQR) of 1.30 (0.90, 2.20) mmol/L and 1.30 (1.00, 2.03) mmol/L, respectively (*p* = 0.002). (9) Bicarbonate: The bicarbonate level of non-survivors with sepsis admitted to the ICU was significantly lower than that of the control group, with medians (IQR) of 22.00 (19.00, 25.00) mmol/L and 23.00 (19.00, 27.00) mmol/L, respectively (*p* = 0.036). (10) Acute Physiology Score (APS): The APS of non-survivors with sepsis admitted to the ICU was significantly higher than that of the control group, with medians (IQR) of 43.00 (35.00, 56.00) and 37.00 (29.00, 51.00), respectively (*p* = 0.015) (Table 1).

**Table 1:** Baseline characteristics of ICU death of IBD complicated with sepsis in the EICU (2014–2015).

Variable	Overall	Sepsis	Non-sepsis	*p*-value^b^
	*N* = 190^a^	*N* = 55^a^	*N* = 135^a^	
Age (years)	56.50 (38.00, 70.00)	58.00 (42.00, 69.00)	54.00 (37.00, 71.00)	0.66
Age (%)				0.44
Old	85 (44.74%)	27 (49.09%)	58 (42.96%)	
Young	105 (55.26%)	28 (50.91%)	77 (57.04%)	
Gender				0.43
Female	102 (53.68%)	32 (58.18%)	70 (51.85%)	
Male	88 (46.32%)	23 (41.82%)	65 (48.15%)	
Race				0.52
African American	23 (12.11%)	5 (9.09%)	18 (13.33%)	
Asian	1 (0.53%)	0 (0.00%)	1 (0.74%)	
Caucasian	152 (80.00%)	45 (81.82%)	107 (79.26%)	
Hispanic	7 (3.68%)	4 (7.27%)	3 (2.22%)	
Native American	1 (0.53%)	0 (0.00%)	1 (0.74%)	
Other/unknown	6 (3.16%)	1 (1.82%)	5 (3.70%)	
Height (cm)	1.68 (1.60, 1.75)	1.65 (1.60, 1.73)	1.68 (1.63, 1.75)	0.13
Weight (kg)	74.10 (62.00, 89.70)	77.80 (63.50, 97.24)	72.20 (61.00, 86.80)	0.014
BMI (kg/m^2^)	26.07 (22.06, 31.31)	30.39 (23.02, 35.68)	25.04 (21.79, 29.76)	<0.001
BMI_Group (%)				<0.001
Low (BMI < 18.5 kg/m^2^)	19 (10.00%)	1 (1.82%)	18 (13.33%)	
Normal (18.5 ≤ BMI < 25 kg/m^2^)	67 (35.26%)	15 (27.27%)	52 (38.52%)	
Overweight (25 ≤ BMI < 30 kg/m^2^)	44 (23.16%)	9 (16.36%)	35 (25.93%)	
Obesity (BMI ≥ 30 kg/m^2^)	60 (31.58%)	30 (54.55%)	30 (22.22%)	
DBP (mmHg)	64.50 (54.00, 75.00)	55.00 (48.00, 69.00)	66.00 (55.00, 76.00)	0.002
SBP (mmHg)	112.50 (97.00, 131.00)	103.00 (88.00, 124.00)	114.00 (101.00, 136.00)	0.005
Heart rate (bpm)	91.00 (82.00, 108.00)	90.00 (80.00, 108.00)	91.00 (83.00, 108.00)	0.83
Respiratory rate (bpm)	19.00 (16.00, 24.00)	20.00 (17.00, 26.00)	19.00 (15.00, 23.00)	0.027
SpO₂(%)	98.00 (96.00, 100.00)	97.00 (94.00, 100.00)	98.00 (96.00, 100.00)	0.087
Temperature (°C)	36.80 (36.50, 37.10)	37.00 (36.60, 37.50)	36.80 (36.40, 37.10)	0.013
HGB (g/dL)	10.21 (2.15)	9.68 (2.15)	10.42 (2.13)	0.014
Plateletcount (K/mcL)	222.50 (152.00, 312.00)	255.00 (149.00, 324.00)	216.00 (152.00, 306.00)	0.71
Neutrophil percentage (%)	81.53 (73.40, 88.00)	81.55 (73.50, 88.10)	81.50 (73.00, 88.00)	0.75
Lymphocyte percentage (%)	9.80 (5.90, 16.00)	8.00 (4.30, 13.60)	10.00 (6.00, 17.00)	0.031
RDW (%)	15.39 (14.30, 17.40)	15.60 (14.80, 17.60)	15.20 (14.18, 17.20)	0.13
Anion gap (mmol/L)	10.00 (7.00, 13.00)	9.00 (7.00, 12.00)	10.00 (7.00, 14.00)	0.017
Lactate (mmol/L)	1.30 (1.00, 2.20)	1.30 (0.90, 2.20)	1.30 (1.00, 2.03)	0.002
Bicarbonate (mmol/L)	23.00 (19.00, 26.00)	22.00 (19.00, 25.00)	23.00 (19.00, 27.00)	0.036
ICU LOS, days	2.07 (1.15, 3.79)	2.84 (1.23, 5.97)	1.95 (1.10, 3.18)	0.02
APS score^c^	39.00 (31.00, 53.00)	43.00 (35.00, 56.00)	37.00 (29.00, 51.00)	0.015

a Normality of continuous variables was tested using the Shapiro–Wilk test. Continuous variables conforming to a normal distribution are presented as mean ± standard deviation (Mean ± SD), while those with a non-normal distribution are presented as median (interquartile range, IQR). Categorical variables are presented as counts (percentage, *n*/%). All statistical tests were two-tailed, and a *p*-value < 0.05 was considered statistically significant.

b Wilcoxon rank sum test; Pearson’s Chi-squared test; Fisher’s exact test.

^c^The APS sums mild multi-system abnormalities into a total score reflecting overall physiological derangement; thus, high total scores can occur without extreme single-organ dysfunction. The majority of patients in this cohort had multiple mild derangements across physiological systems, consistent with the pattern of multi-organ involvement in critical illness.

BMI, body mass index; DBP, diastolic blood pressure; SBP, systolic blood pressure; SpO₂, peripheral oxygen saturation, HGB, hemoglobin, RDW, red cell distribution width; APS Score, Acute Physiology Score.

### 3.3 Analysis of the relationship between APS and IBD patients with sepsis using weighted logistic regression model

Model 1 was not adjusted for any covariates, demonstrating a significant positive correlation between APS and sepsis in non-survivors admitted to the ICU (OR = 1.021, 95% CI: 1.006–1.038, *p* = 0.008). When APS was grouped by quartiles, the highest quartile (Q4, APS ≥ 53) showed a significantly increased risk of sepsis in non-survivors admitted to the ICU compared to the lowest quartile (Q1, APS < 31), with an OR of 3.744 (95% CI: 1.447–10.655, *p* = 0.009). The trend test indicated a remarkable positive correlation between APS and the risk of sepsis in non-survivors admitted to the ICU (*p* for trend = 0.039).

Model 2 was adjusted for weight and BMI, and the association between APS and the risk of sepsis in non-survivors admitted to the ICU remained significant (OR = 1.023, 95% CI: 1.007–1.041, *p* = 0.007). When APS was grouped by quartiles, the results showed that the risks of sepsis in non-survivors admitted to the ICU continued to increase in Q2 and Q4 compared to Q1, with ORs of 3.532 (95% CI: 1.218–11.298, *p* = 0.025) and 4.900 (95% CI: 1.735–15.488, *p* = 0.004), respectively. At the same time, the trend test confirmed the positive correlation between APS and the risk of sepsis in non-survivors admitted to the ICU (*p* for trend = 0.018).

Model 3 was further adjusted for DBP, SBP, HGB, Lymphocyte percentage, Anion gap, Lactate, Respiratory rate, and Temperature. The association between APS and the risk of sepsis in non-survivors admitted to the ICU remained remarkable and statistically significant (OR = 1.027, 95% CI: 1.009–1.045, *p* = 0.004). When APS was grouped by quartiles, the highest quartile (Q4, APS ≥ 53) exhibited a markedly increased risk of sepsis in non-survivors admitted to the ICU (OR = 4.856, 95% CI: 1.675–15.713, *p* = 0.005), with the second quartile (Q2) also being statistically significant for the risk of sepsis compared with Q1 (OR = 3.449, 95% CI: 1.157–11.374, *p* = 0.032). Additionally, the trend test indicated that even after adjusting for covariates, higher APS levels remained associated with the risk of sepsis in non-survivors admitted to the ICU (*p* for trend = 0.020) (Table 2).

**Table 2:** Association between APS and sepsis in non-surviving IBD patients.

Characteristics	OR	Model 1	*p*-value	OR	Model 2	*p*-value	OR	Model 3	*p*-value
		95%CI			95%CI			95%CI	
APS Score									
APS Score continuous	1.021	(1.006, 1.038)	0.008	1.023	(1.007, 1.041)	0.007	1.027	(1.009, 1.045)	0.004
APS Score quantile									
Q1 (low)	Ref	Ref		Ref	Ref		Ref	Ref	
Q2	3.076	(1.167, 8.854)	0.028	3.532	(1.218, 11.298)	0.025	3.449	(1.157, 11.374)	0.032
Q3	1.571	(0.558, 4.678)	0.398	1.654	(0.527, 5.484)	0.394	1.688	(0.524, 5.763)	0.386
Q4 (high)	3.744	(1.447, 10.655)	0.009	4.900	(1.735, 15.488)	0.004	4.856	(1.675, 15.713)	0.005
*p* for trend			0.039			0.018			0.020

Results of odds ratios (OR values, 95% confidence interval, 95% CI) along with corresponding *p*-values.

OR, odds ratio; CI, confidence intervals; APS, Acute Physiology Score.

APS: Q1: APS < 31; Q2: 31 ≤ APS < 39; Q3: 39 ≤ APS < 53; Q4: APS ≥ 53.

The Model 1 was the crude model.

The Model 2 was adjusted by weight, BMI.

The Model 3 was adjusted by weight, BMI, DBP, SBP, HGB, lymphocyte percentage, anion gap, lactate, respiratory rate, temperature.

### 3.4 Exploring influencing factors of sepsis in IBD patients admitted to the ICU via XGBoost model and machine learning

**Figure 2:**
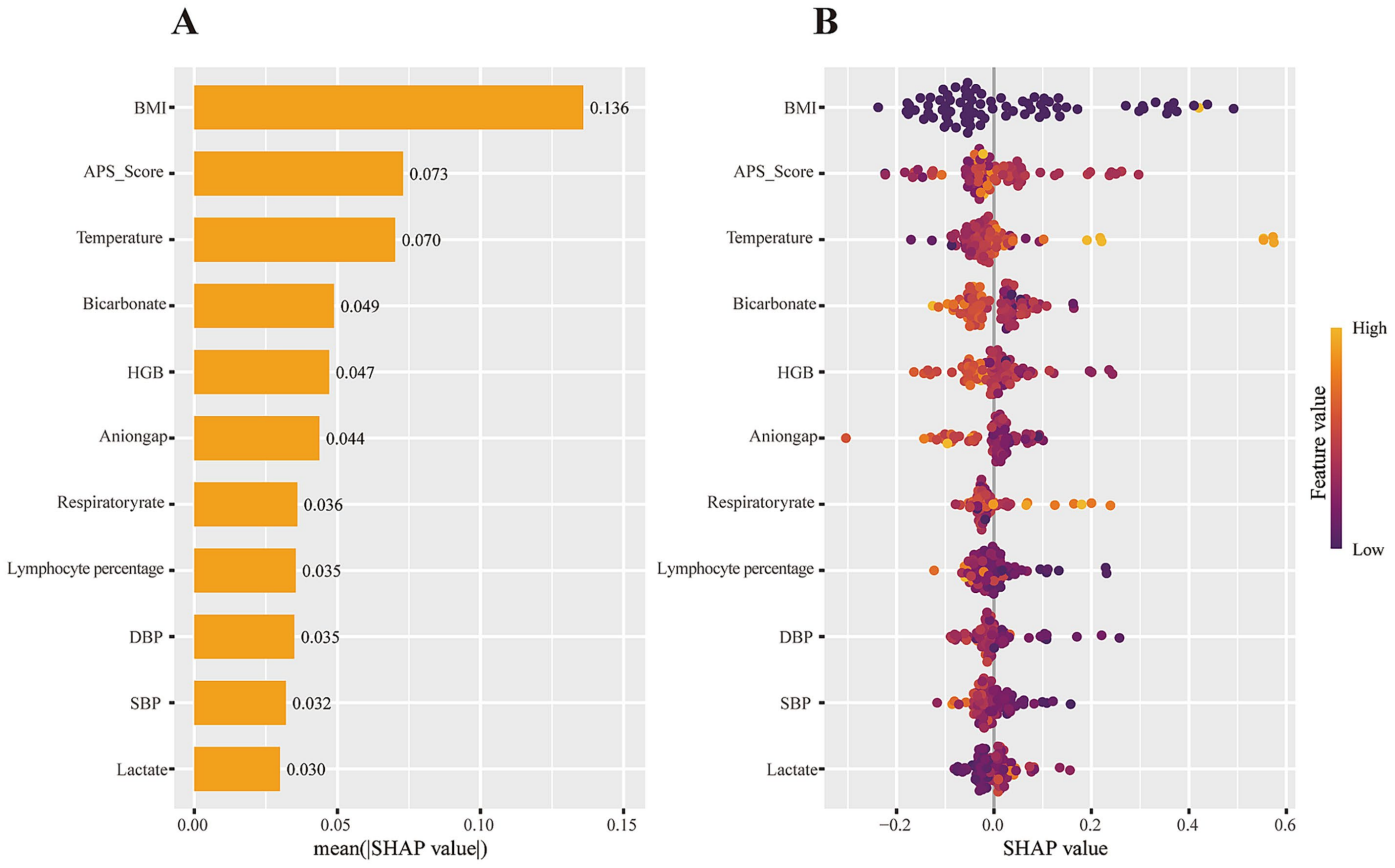
Feature importance for predicting sepsis in non-survivors using the XGBoost model. **(A)** Bar chart of the mean absolute SHAP values for the top predictors. **(B)** SHAP summary plot depicting the impact and distribution of each feature on the model output.

In this study, the XGBoost model was used to evaluate the contribution of multiple variables to the risk of death in patients with IBD complicated with sepsis, and model interpretation was performed using the SHapley Additive exPlanations (SHAP) framework. The variable importance ranking based on mean absolute SHAP values (mean |SHAP|) is shown in Figure 2A. Body mass index (BMI) was identified as the feature with the highest predictive contribution (mean |SHAP| = 0.136); Acute Physiology Score (APS) was the second most important predictor (mean |SHAP| = 0.073), followed by body temperature (mean |SHAP| = 0.070), bicarbonate (mean |SHAP| = 0.049), hemoglobin (mean |SHAP| = 0.047), and anion gap (mean |SHAP| = 0.044). SHAP dependence plots illustrated the directional relationships between these features and the predicted risk of sepsis in IBD patients (Figure 2B): higher values of APS, anion gap, body temperature, respiratory rate, and BMI were associated with an increased risk of sepsis; conversely, higher levels of bicarbonate and hemoglobin were associated with a decreased risk. Owing to the small sample size, the XGBoost and SHAP analyses were exploratory and intended for hypothesis generation. Due to the small sample size (*N* = 190, 55 sepsis events), the SHAP-based feature importance rankings are inherently unstable, and quantitative assessments of uncertainty are not reported. These rankings should not be overinterpreted and require validation in larger, unselected cohorts.

### 3.5 Exploring the nonlinear relationship between APS and ICU-non-survivors with sepsis using RCS curves

**Figure 3:**
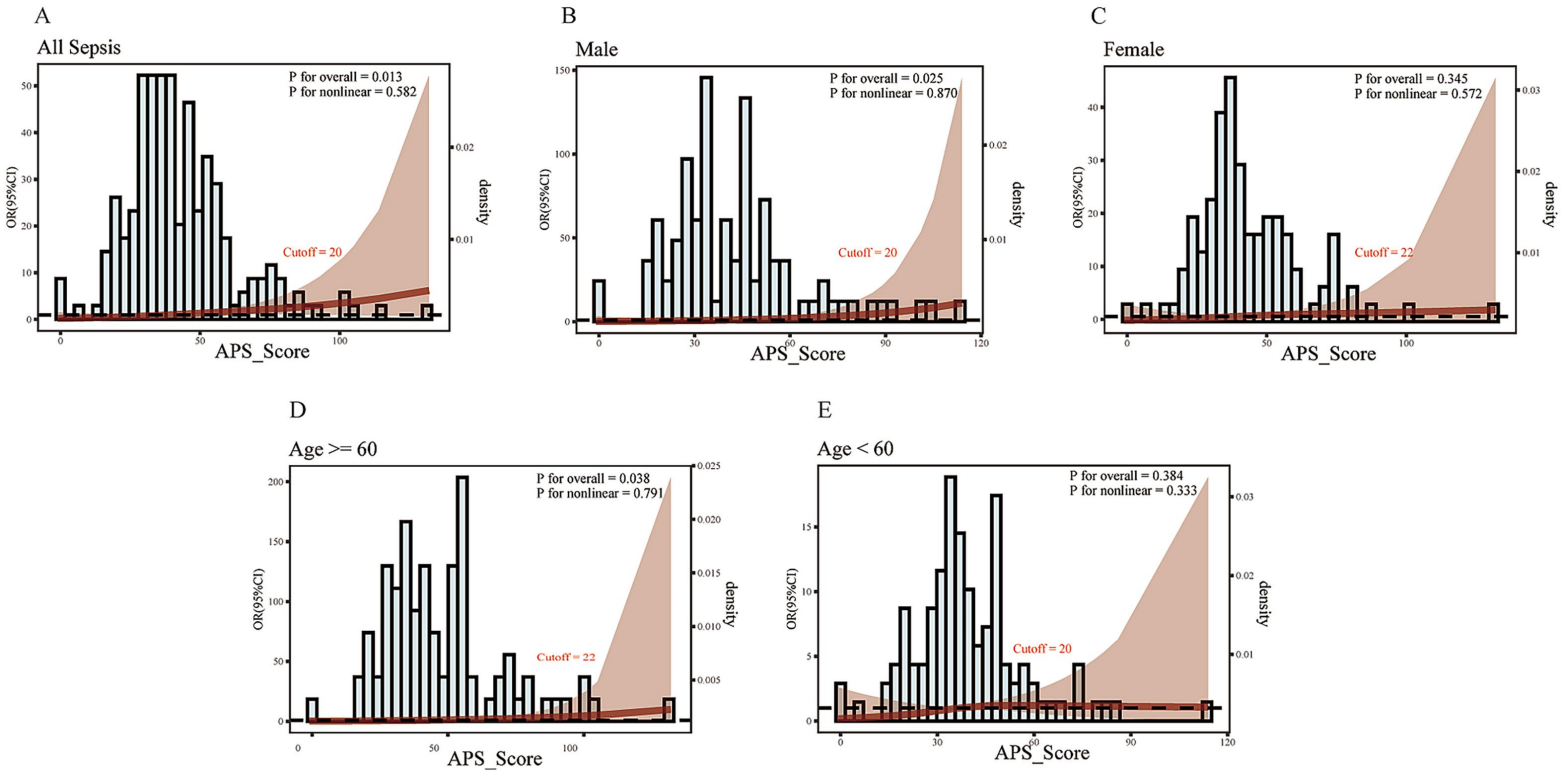
Restricted cubic spline curves showing the association between Acute Physiology Score and the risk of sepsis in non-survivors. **(A)** all sepsis patients, **(B)** male subgroup, **(C)** female subgroup, **(D)** age ≥ 60 years subgroup, **(E)** age < 60 years subgroup.

The relationship between APS and sepsis in non-survivors admitted to the ICU was assessed using RCS curves, adjusting for all relevant covariates. The results showed that: (1) Linear Trend: There is a significant linear trend between APS and sepsis in non-survivors admitted to the ICU (*p* for overall = 0.013), with no significant nonlinear relationship observed (*p* for nonlinear = 0.582). The RCS curve indicated that when APS reached or exceeded 20, the risk of sepsis in non-survivors admitted to the ICU significantly increased, with the 95% CI gradually widening (Figure 3A). (2) The male group (Figure 3B) showed a significant association between APS and sepsis in non-survivors admitted to the ICU (*p* for overall = 0.025), without any nonlinear trend (*p* for nonlinear = 0.870). By contrast, there was no significant linear relationship between APS and sepsis in non-survivors admitted to the ICU in the female group (*p* for overall = 0.345) (Figure 3C), and no significant nonlinear trend was observed (*p* for nonlinear = 0.572). (3) In the age group (≥60 years old) (Figure 3D), a remarkable association was found between APS and sepsis in non-survivors admitted to the ICU (*p* for overall = 0.038), with no nonlinear trend (*p* for nonlinear = 0.791). When APS reached 22, the risk of sepsis in non-survivors admitted to the ICU was substantially elevated. In comparison, the age group (<60 years old) demonstrated no significant linear relationship between APS and sepsis in non-survivors admitted to the ICU (*p* for overall = 0.384), with no significant nonlinear trend observed (*p* for nonlinear = 0.333) (Figure 3E). The total sample size of this study was 190 (IBD patients who died during ICU admission). After stratification by sepsis status (55 with sepsis, 135 without sepsis), certain subgroups had relatively limited sample sizes. This sample size is primarily constrained by the actual number of “IBD with ICU mortality” cases recorded in the eICU-CRD database (the 2014–2015 version contains 190 cases meeting the inclusion criteria). Although this does not reach the ideal size for large-sample statistics, it provides adequate data support for association analyses in this rare critically ill subgroup. The wide 95% confidence intervals in certain APS ranges reflect limited stability attributable to larger standard errors in small-sample data and should be interpreted with caution.

### 3.6 The relationship between APS and baseline characteristics among subgroups

**Figure 4:**
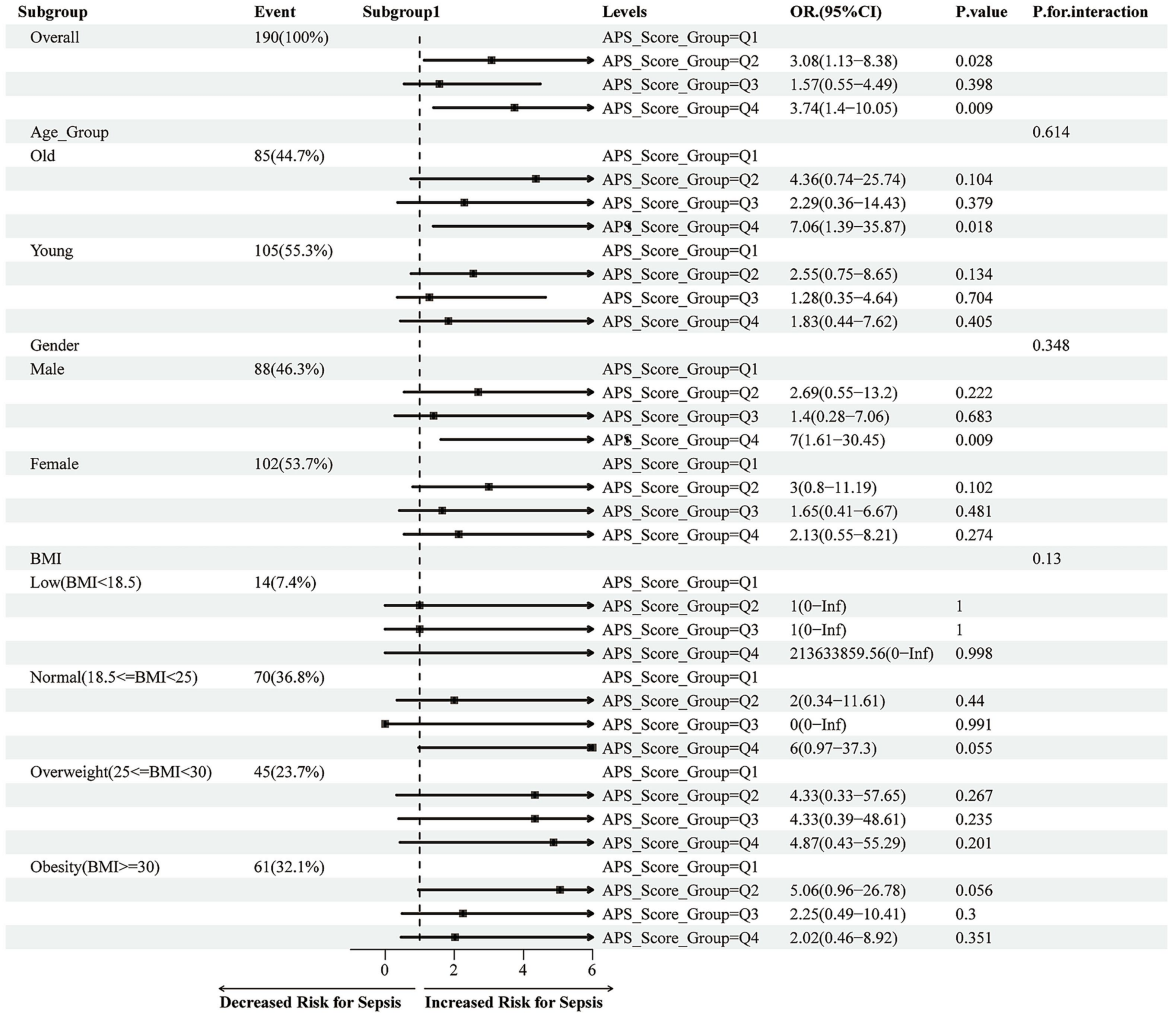
Forest plot of the association between Acute Physiology Score (APS) quartiles and sepsis, stratified by baseline characteristics. The underweight BMI subgroup showed an extreme, unstable odds ratio due to sparse data and complete separation; this estimate is not reliable and should not be interpreted.

The association between APS and sepsis in non-survivors was analysed using weighted logistic regression. Subgroup analyses were conducted across various baseline characteristics (Figure 4). No significant interaction effects were observed in any subgroups. However, stratified analyses revealed a pronounced association in specific populations: among elderly patients (≥60 years), the highest APS quartile (Q4) was significantly associated with sepsis (OR = 7.06, 95% CI: 1.39–35.87, *p* = 0.018), while in males, the high-APS group (Q4 vs. Q1) also showed a markedly increased risk (OR = 7.00, 95% CI: 1.61–30.45, *p* = 0.009). Notably, the underweight BMI subgroup showed an extreme and unstable OR estimate due to sparse data and complete separation; this result should not be interpreted. All subgroup analyses are exploratory and hypothesis-generating due to small sample size and sparse data.

### 3.7 Exploring potential mechanistic links

Additionally, the relationship between APS and biochemical indicators after adjusting for gender, race, height, weight, temperature, and respiratory rate was also assessed in this study, indicating a statistically significant positive correlation between APS and anion gap (*β* = 0.047, 95% CI: −0.003 to –0.098, *p* = 0.044) and lactate (*β* = 0.235, 95% CI: 0.04–0.44, *p* = 0.018) and a statistically significant negative correlation between APS and bicarbonate (*β* = −0.102, 95% CI: −0.153 to −0.053, *p* < 0.001), as shown in Table 3. To further eliminate bias from the overall population regarding the results of sepsis in non-survivors admitted to the ICU, the correlation between APS and biochemical indicators in the control group was also assessed, in which the results for anion gap, lactate, and bicarbonate were consistent with those of the entire population. The association between APS score and lactate, a key marker of acute physiological stress independent of the APS components, was further analyzed in the overall population and subgroups. As shown in Table 3, APS score was significantly positively correlated with lactate in the overall population (*β* = 0.24, 95% CI: 0.04–0.451, *p* = 0.019). This significant positive correlation remained consistent in both the sepsis group (*β* = 0.265, 95% CI: −0.018 to –0.593, *p* = 0.036) and the non-sepsis group (*β* = 0.289, 95% CI: 0.007–0.58, *p* = 0.044). The stable association across different subgroups suggests that higher APS scores are robustly linked to elevated lactate levels, which reflects the severity of tissue hypoperfusion and acute physiological derangement in critically ill patients.

**Table 3:** The association between APS_Score and sepsis related Indicators in overall population and controls.

Mediating factors	Overall (*n* = 190)			Sepsis patients (*n* = 55)			Non-sepsis patients (*n* = 135)		
	*β* value	95% CI	*p*-value	*β* value	95% CI	*p*-value	*β* value	95% CI	*p-*value
Aniongap	0.054	(0.008, 0.101)	0.021	0.213	(0.089, 0.351)	0.001	0.043	(−0.007, 0.095)	0.029
Lactate	0.24	(0.04, 0.451)	0.019	0.265	(−0.018, 0.593)	0.036	0.289	(0.007, 0.58)	0.044
Bicarbonate	−0.118	(−0.171, −0.068)	*p* < 0.001	−0.156	(−0.27, −0.05)	0.004	−0.116	(−0.177, −0.058)	*p* < 0.001

Results of regression analyses are presented as beta values (95% confidence interval, 95% CI) along with corresponding *p*-values.

### 3.8 Sensitivity analysis

Sensitivity analyses confirmed the robustness of our primary findings. Temperature and respiratory rate were selected for exclusion because they are core components of the APS; removing them avoids information redundancy between the total APS score and its individual components, which would otherwise introduce overadjustment bias and distort the estimated association. This reverse validation strategy confirms that the observed association is not driven by any single component but by the integrative value of the APS in capturing multidimensional physiological derangements. The association between APS and sepsis remained significant after excluding temperature (Supplementary Table S1), with consistent effect sizes in both unadjusted (OR = 1.021, 95% CI: 1.006–1.038, *p* = 0.008) and adjusted models (OR = 1.022, 95% CI: 1.005–1.040, *p* = 0.013). After further excluding respiratory rate, the association also persisted: in the unadjusted model, OR = 1.021, 95% CI: 1.006–1.038, *p* = 0.008; in the adjusted model, OR = 1.027, 95% CI: 1.009–1.045, *p* = 0.004 (Supplementary Table S2).

## 4 Discussion

Sepsis represents a significant health challenge for IBD patients admitted to the ICU, and its early identification is critical for improving prognosis. APS quantifies physiological disturbance by integrating vital signs and laboratory parameters—many of which overlap with early sepsis manifestations—making it a relevant tool for characterizing physiological severity in this critically ill subgroup (19, 20). IBD patients face heightened infection risks due to intestinal barrier disruption, bacterial translocation, and immunosuppressive therapies (21, 22). Sepsis can further exacerbate intestinal injury, creating a vicious cycle (23, 24). APS also captures metabolic and electrolyte imbalances common in IBD, such as anemia and malnutrition, which may increase sepsis susceptibility (25).

A significant linear trend was observed between APS and sepsis risk, supporting its role in continuous risk assessment. Moreover, quartile analysis identified APS ≥ 53 (Q4) as a high-risk threshold, simplifying clinical triage and resource allocation. As this value was derived from the top quartile of a non-survivor population, it is not validated for general use in the overall ICU-IBD population and should be interpreted with extreme caution. This was an exploratory cutoff based on weighted quartiles; performance metrics including sensitivity, specificity, and predictive values were not calculated given the retrospective, small, and highly selected study design, which limits its clinical utility. The XGBoost model identified APS as a top predictor of sepsis, alongside BMI, temperature, and respiratory rate. While BMI emerged as an important predictor in this cohort, this finding requires careful interpretation. Elevated BMI in IBD patients may reflect long-term corticosteroid use, which is associated with both weight gain and elevated infection risk, suggesting that BMI could serve as a surrogate marker of immunosuppressive exposure. In addition, high BMI may also be driven by fluid overload, metabolic disturbances, or chronic comorbidities rather than adiposity alone. Of note, low BMI may indicate malnutrition and frailty, which are well-established risk factors for adverse outcomes in critical illness. Therefore, the association between BMI and sepsis risk may not be purely linear and could potentially follow a U-shaped pattern, wherein both high and low BMI values may confer elevated risk in this vulnerable population. This finding further highlights the need to incorporate IBD-specific treatments, disease activity, complications, and comorbidities into future predictive models (26, 27).

Notable conceptual circularity exists between APS and sepsis, as APS comprises 12 physiological variables (including temperature, respiratory rate, arterial pH, and bicarbonate) that overlap substantially with SIRS criteria and Sepsis-3 definitions. Thus, the observed association may be partly tautological rather than reflecting independent predictive ability. Although the association persisted in Model 3 and sensitivity analyses after adjusting for several overlapping components, this does not fully eliminate shared physiological signal or conceptual overlap.

Subgroup analyses revealed that the APS–sepsis association was stronger in males and elderly patients (≥60 years). While immunosenescence, comorbidities, and sex-based differences in immune response may plausibly contribute to this pattern, these explanations remain hypothetical and are not directly supported by the current data; further validation in targeted studies is warranted (28–30). APS was also correlated with elevated anion gap and lactate, and reduced bicarbonate—indicators of metabolic acidosis commonly seen in sepsis (31, 32). These relationships may partly explain how APS captures sepsis-related physiological derangements (33). Several limitations should be noted. First, by restricting the cohort to non-survivors, we introduced collider bias because we conditioned on death, as we condition on death (strongly associated with both APS and sepsis). Therefore, our findings cannot be generalized to all ICU-admitted IBD patients, nor do they support clinical prediction. Additionally, the temporal relationship between APS and sepsis remains unclear because we did not adopt a cohort design with death as a censoring event. The median ICU stay of approximately 2 days suggests most deaths occurred shortly after admission; due to database limitations, we cannot distinguish whether sepsis was present at admission or developed during the stay. If sepsis was already present at admission, the observed association may partly reflect reverse causality. Furthermore, IBD identification relied only on ICD-9 codes 555.9 and 556.9, as the eICU-CRD database uniformly records only these two unspecified codes, precluding finer subtype stratification. Sepsis was ascertained using ICD-9 codes (038.9, 995.90, 995.91, 995.92, and 785.52) supplemented by comorbidity diagnoses, but administrative coding may still introduce sensitivity and specificity biases. Second, Model 3 includes variables that are APS components or downstream physiological markers; although VIF values were unremarkable and results remained consistent across models, this may still constitute conceptual overadjustment and produce conservatively biased estimates. This concern is distinct from the general principle of knowledge-driven covariate selection (18), which guided our variable choice but does not address the inclusion of exposure-defining or mediator-like variables. Third, the small sample (*n* = 190) and few sepsis events (*n* = 55) yield insufficient statistical power for complex analyses, risking unstable and unreliable estimates. Sparse data led to an extreme, uninterpretable OR in the underweight BMI subgroup. Furthermore, due to the recording patterns of the eICU-CRD (2014–2015 version), key intervention variables such as mechanical ventilation, vasoactive agents, and renal replacement therapy had high proportions of missing data, and standardized ICU length-of-stay could not be reliably computed, precluding their inclusion in adjusted analyses and leaving potential residual confounding. Fourth, key IBD-specific confounders, including disease activity and treatments such as corticosteroids, immunomodulators, and biologics, were unavailable. These are major determinants of infection and sepsis risk in IBD, and their omission may substantially bias the observed association. Finally, the XGBoost analysis was exploratory in nature and limited by the small sample size; feature importance rankings (e.g., the high importance of BMI) may be unstable and warrant confirmation in larger, externally validated cohorts.

## 5 Conclusion

In this cohort of 190 non-survivors, APS was independently associated with sepsis risk in critically ill IBD patients. Correlative analyses suggested potential links between APS and metabolic acidosis markers. However, given the small, selected sample and inherent study limitations, these findings are hypothesis-generating and should not be interpreted as supporting clinical prediction. Further validation in larger, unselected cohorts is required.

## Data Availability

The raw data supporting the conclusions of this article will be made available by the authors, without undue reservation.
